# Hypertriglyceridemia–Induced Acute Pancreatitis in a Patient With Type 2 Diabetes Mellitus

**DOI:** 10.7759/cureus.9414

**Published:** 2020-07-27

**Authors:** Wafa A Aldhaleei, Abdulaziz Alnuaimi, Akshaya S Bhagavathula

**Affiliations:** 1 Gastroenterology, Sheikh Shakhbout Medical City, Abu Dhabi, ARE; 2 Gastroenterology, Abu Dhabi Health Services Company (SEHA), Abu Dhabi, ARE; 3 Public Health, Institute of Public Health, College of Medicine and Health Sciences, United Arab Emirates University, Al Ain, ARE

**Keywords:** severe pancreatitis, lipid metabolism, insulin injection, triglycerides, lipase, 1.hepatology 2. diabetes 3. neurology 4. tuberculosis 5. infectious diseases, types 2 diabetes, middle east north africa region, familial lipid disorders, pancreas disease

## Abstract

Acute pancreatitis (AP) is an uncommon potential complication of severe hypertriglyceridemia (HTG). We reported a case of a 45-year-old man admitted with HTG-induced AP (HTG-AP). The patient was a known diabetic (glycated hemoglobin levels: 9.5%), his triglycerides level was 3587.2 mg/dl, and the lipase level was 242 IU/L. A CT scan revealed AP. The patient was treated with a low-dose insulin infusion (0.05 unit/kg/hr) with dextrose for six days. His triglycerides came down to 673.1 mg/dl, and he was discharged. Further investigations are needed to understand the efficacy of low-dose insulin in the management of HTG-AP.

## Introduction

Familial hypertriglyceridemia (FHTG) is a genetic disorder caused by mutations in five genes (LPL, APOC2, APOA5, LMF1, and GPIHBP1) that regulate plasma lipid metabolism [1-3]. Elevated serum triglycerides (TG) and chylomicrons in these patients increase the plasma viscosity leading to tissue ischemia and inflammation [4]. Eruptive xanthoma associated with hypertriglyceridemia (HTG), occurs either as a result of primary (familial) or secondary causes such as poorly controlled diabetes, elevated body mass index (BMI), or alcohol abuse, or a combination of both [2]. In some patients, a triad of diabetic ketoacidosis (DKA), HTG, and acute pancreatitis (AP) were also described [3,5]. Therefore, early recognition can prevent the progression of disease and aid aggressive treatment to minimize the complications associated with FHTG [2].

AP is a potentially life-threatening complication of severe HTG. The risk and severity of AP increase with the increasing levels of serum TG. Several studies have characterized hypertriglyceridemia-induced acute pancreatitis (HTG-AP) patients with abdominal pain, raised pancreatic enzymes over three times than the normal level, and radiological imaging suggestive of AP [4,6]. Multiple treatment modalities have been suggested for patients with HTG-AP, such as permanent removal of TG by plasmapheresis, but the results are conflicting [7]. Other treatments with limited supporting data incorporate the use of insulin and heparin to enhance lipoprotein lipase activity [6,8]. Here, we present a case of HTG-AP treated with low dose insulin infusion.

## Case presentation

A 45-year-old man, known to have diabetes mellitus type 2 and FHTG (diagnosed based on genetic testing) from past six years, not compliant with his treatment (atorvastatin 10 mg bedtime and metformin 500 mg twice daily), presented to a tertiary hospital with a one-day history of abdominal pain and three episodes of vomiting. The patient had experienced similar symptoms six years ago. He denied eating food from a restaurant, although he reported that his diet is high in carbohydrates and fat. On clinical examination, abdominal tenderness was elicited, and no eruptive xanthomas were present. His blood test result on the day of admission was not reported as it was highly lipemic. Pertinent laboratory tests showed: lipase 242 IU/L (reference: 13-60), amylase 41 IU/L (reference: 28-100), albumin 33 g/L (reference: 35-52), and creatinine 40 micromol/L (reference: 62-106). Aspartate aminotransferase (AST) and alanine aminotransferase (ALT) were not recorded as the sample was lipemic. Total cholesterol was 592.8 mg/dl (reference: 150.8-201); high-density lipoprotein (HDL), 15.8 mg/dl (reference: 42.5-61.8); and triglycerides, 3587.2 mg/dl (reference: 44.2-150.5). Low-density lipoprotein (LDL) was not recorded. HbA1c was 9.7% (reference: 4.6-6.2). His venous blood gas showed a pH of 7.42 (reference: 7.35-7.45) and HCO3 of 25 mmol/L (reference: 22-26). He had a CT scan without contrast on the day of admission, which was suggestive of AP.

**Figure 1:**
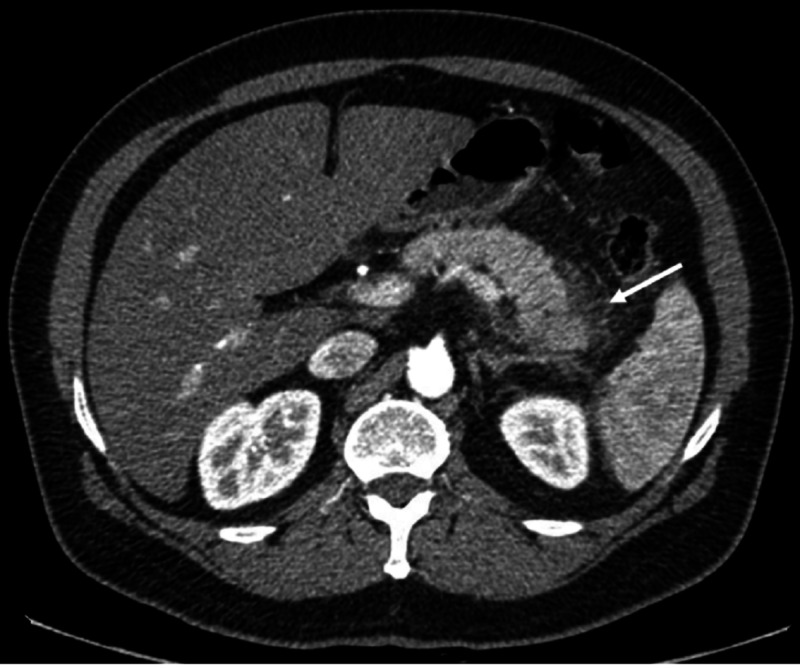
CT abdomen with contrast on day 3 CT scan showing features of pancreatitis and peripancreatic inflammation (arrow)

The patient was diagnosed with HTG-AP. On day 1, the patient was kept nil per mouth and started on a continuous infusion of normal saline at 135 ml/hr for 24 hours. On day 2, he was reviewed by the endocrinologist and recommended starting IV insulin infusion 4 units/hr and dextrose normal saline IV fluids until the TG level falls below 443 mg/dl. Besides, atorvastatin (40 mg once daily (OD)), fenofibrate (145 mg OD), omega-3 (1 g OD), and aspirin (100 mg OD) were given. On day 3, his abdominal pain resolved, and the CT scan with contrast revealed acute pancreatitis (Figure 1). On day 6, his lipase level reduced to normal (40 IU/L), and TG level came down to 673.1 mg/dl; he was discharged.

On discharge, the patient was kept on insulin glargine 40 units bedtime, insulin NovoRapid® four units three times daily with 1 g metformin twice daily. All oral medications (atorvastatin, fenofibrate, omega-3 fatty acids, and aspirin) were continued. Four days after discharge, the patient was seen in the clinic by the endocrinologist. His lipase levels were normal (21 IU/L), and TG was at 549.1 mg/dl.

## Discussion

While AP has multiple etiologies, the underlying cause of HTG-AP in our patient is due to poorly controlled type 2 diabetes (HbA1c: 9.7%). The exact mechanism by which HTG induces AP in patients with diabetes has not been fully elucidated. Studies reported that insulin resistance in patients with type 2 diabetes increases the production of TG and reduces its plasma clearance [4].

Diagnosing HTG-AP can be challenging due to in vitro interference between high TG levels above 500 mg/dl and lipase activities. To the best of our knowledge, this is the first case report to identify a high level of TG of 3587.2 mg/dl, with moderately elevated lipase levels of 242 IU/L. A case report published by Gan et al. presented an HTG-AP patient with a TG level of 3410 mg/dl and lipase of 754 IU/L [9]. A recent cohort study on 716 AP patients found that the risk of severe AP was strongly associated with TG levels over 2000 mg/dl [10]. In these patients with high TG levels, over 3543 mg/dl, most are treated with plasmapheresis or glucose-heparin-insulin (GLU-HEP-INS) [10].

Definitive guidelines for the treatment of HTG-AP have not yet been established [11]. Prior case reports managed their patients with insulin infusion with dextrose, heparin, plasmapheresis, and different therapeutic plasma exchange methods were used [8,12,13]. Garg et al. and Twilla et al. used an insulin infusion of 0.1 units/kg/hr with dextrose and heparin to treat HTG-AP [6,7]. However, in our case, the patient showed a response to low-dose insulin infusion of 0.05 units/kg/hr with dextrose for six days, resulting in a reduction in the TG levels by 2914 mg/dl and lipase by 202 IU/L.

In general, the clinical presentation of HTG-AP includes epigastric pain, nausea, vomiting, and signs of eruptive xanthomas due to high TG levels. On clinical examination, our patient did not have xanthomas but had abdominal pain and vomiting of one-day duration. Eruptive xanthomas is a predictive sign of familial and secondary HTG complications, like pancreatitis [2]. In patients with a lack of xanthomas, like ours, an HTG-AP diagnosis can be delayed.

## Conclusions

HTG-AP is a serious condition requiring swift diagnosis and treatment. In our case, HTG-AP was managed with low-dose insulin infusion with dextrose for six days that prevented the progression of the pancreatitis-related complications. Further investigations are needed to understand the efficacy of low-dose insulin in the management of HTG-AP.
